# Fast and Sensitive Measurement of Off‐Flavors in Recirculating Aquaculture Systems

**DOI:** 10.1002/rcm.70085

**Published:** 2026-05-01

**Authors:** Pedro Martínez Noguera, Sylvester Holt, Raju Podduturi, Wender L. P. Bredie, Jonathan D. Beauchamp, Mikael A. Petersen

**Affiliations:** ^1^ Design and Consumer Behaviour, Department of Food Science University of Copenhagen Frederiksberg Denmark; ^2^ Department of Sensory Analytics and Technologies Fraunhofer‐Institute for Process Engineering and Packaging IVV Freising Germany

**Keywords:** 2‐methylisoborneol, direct injection, geosmin, headspace, off‐odor, PTR‐MS

## Abstract

**Rationale:**

Geosmin and 2‐methylisoborneol (2‐MIB) are odor‐active compounds responsible for earthy off‐flavors in fish, creating significant quality concerns in recirculating aquaculture systems (RAS). Their extremely low odor thresholds make rapid and highly sensitive detection essential for maintaining product quality and consumer acceptance in aquaculture production.

**Methods:**

A novel analytical method was developed using static headspace sampling combined with proton transfer reaction‐mass spectrometry (PTR‐MS) for the detection of geosmin and 2‐MIB in aquaculture water. Sample preparation was optimized by sodium chloride addition to enhance *salting out*, incubation at 90 °C, and maximization of headspace injection speed into the PTR‐MS system to improve analyte sensitivity.

**Results:**

The optimized method achieved detection limits of 19 ng·L⁻¹ for geosmin and 4 ng·L⁻¹ for 2‐MIB with a total sample screening time of less than 10 minutes. Detection limits were within the same low ng·L⁻¹ range as the odor thresholds of these compounds, enabling quantification at sensorily relevant concentrations associated with off‐flavor perception.

**Conclusions:**

This PTR‐MS‐based approach provides a rapid and sensitive method for objective detection of geosmin and 2‐MIB in aquaculture water at concentrations relevant to sensory off‐odor impairment. The method shows strong potential for routine monitoring in RAS and may be extended to other volatile compounds and aqueous analytical applications.

## 1 Introduction

The semi‐volatile terpenoid‐based compounds geosmin and 2‐methylisoborneol (2‐MIB) are of significant detriment to the aquaculture industry, the fastest‐growing food production sector currently worldwide [3, 5]. Geosmin and 2‐MIB, among other compounds, can cause unpleasant off‐flavors in aquaculture fish that are described as “earthy” and “musty” [3, 4] and lead to consumer rejection. Recirculated aquaculture systems (RAS) are particularly affected by this problem, with repercussions on fish marketability and an associated negative impact on the economic sustainability of fish farms [5]. Both compounds, which cannot be synthesized by the fish themselves and are thus of exogenous origin, have been detected in many RAS‐farmed fish species and are known to be produced by diverse aquatic microorganisms, including Actinobacteria, Cyanobacteria, and Myxobacteria (Myxococcota) [3]. When present in aquaculture water, these lipophilic semi‐volatile terpenoid alcohols can accumulate in the fatty tissue of the animals and elicit undesired off‐flavor impressions [6]. It is therefore crucial for the aquaculture industry to have at its disposal a robust and reliable analytical method to monitor these compounds to quickly and efficiently identify potential off‐flavor problems in their products. From an operational perspective, the development of an online analytical tool capable of dynamically monitoring these compounds during production or depuration would be highly beneficial for the sector.

The identification and quantification of these compounds in water has been hitherto reported only for chromatographic methods, predominantly gas chromatography (GC) but also liquid chromatography (LC) [7]. A broad range of preconcentration techniques have been used, such as solid phase extraction (SPE; [8]), stir‐bar sorptive extraction (SBSE; [9]), or liquid–liquid extraction (LLE; [10]), which are typically followed by GC separation and mass spectrometric (MS) detection. GC–MS represents the benchmark analytical technique for volatile organic compound (VOC) identification and quantification due to its robustness and its capability for unequivocal compound identification. Most of the preconditioning steps necessary for GC–MS, however, are labor‐intensive, time‐consuming, and requiring skilled personnel, and often large quantities of solvents [11]. There is therefore an interest in developing more cost‐effective, rapid, simpler, and direct analytical methods to detect low quantities of off‐flavors in aquaculture water to increase sample throughput and offer a timely detection as a quality assurance measure in this sector.

This study proposes a novel approach for a targeted analysis of geosmin and 2‐MIB in water based on the direct injection of static headspace from the incubated sample using proton‐transfer‐reaction mass spectrometry (PTR‐MS). The sensitivity of the method is especially important due to the extremely low odor thresholds (OTs) of these off‐odor compounds, reported to be 3.8 ng·L^−1^ for geosmin and 15 ng·L^−1^ for 2‐MIB in water [12]. PTR‐MS is a well‐established direct‐injection technology for real‐time analyses of VOCs that offers high sensitivity and fast analytical response [16, 17]. Its operating principles are described in the literature [18, 23] and are therefore only summarized here. Unlike GC–MS, which relies on chromatographic separation and compound fragmentation via highly energetic electron ionization (EI; typically at 70 eV) for chemical structure elucidation, PTR‐MS uses hydroxonium ions (H_3_O^+^) to protonate VOCs via soft chemical ionization (CI; typically 1–2 eV), with the ensuing ions detected by time‐of‐flight (TOF) mass spectrometry [17]. In PTR‐MS there is no physical compound separation prior to detection, with VOCs directly injected and ionized in the reaction chamber (typically 100 ms from sampling to detection). The high resolution of the TOF‐MS detection system allows discrimination of nominally isobaric compounds, although structural isomers cannot be differentiated without prior GC separation [13].

This paper reports on a method development and corresponding systematic study of the optimal detection of geosmin and 2‐methylisoborneol in aqueous solutions by PTR‐MS. The optimized method was compared analytically with a benchmark GC–MS method for the quantitation of geosmin in a set of RAS water samples. To our knowledge, this work represents the first investigation of the detection and quantitation of geosmin and 2‐MIB in water by PTR‐MS.

## 2 Materials and Methods

### 2.1 Chemicals

Pure reference standards of geosmin (≥ 97%) and 2‐methylisoborneol (≥ 98%) (both procured from Sigma Aldrich, Steinheim, Germany) were used for initial detectability and fragmentation studies. Certified chemical reference standards in methanol (Sigma Aldrich, Steinheim, Germany) were used for the headspace optimization study. Reference standards in methanol (but also the pure ones) were diluted in water to avoid detector saturation and signal suppression (due to high concentrations of methanol) and therefore optimize instrumental performance. Details of the physicochemical properties of the two compounds are given in Table 1. Sodium chloride (NaCl, 99.5%; ThermoFisher Scientific, Denmark) was used for the headspace optimization experimental design and the final PTR‐MS measurements.

**TABLE 1:** Physicochemical properties of geosmin and 2‐methylisoborneol.

Common name	Elemental composition	MW [g·mol^−1^]	Log P/K_ow_ ^a^	K_h_ [atm m^3^·mol^−1^]^b^	Boiling point [°C]^c^	Vapor pressure [atm]^b^	Aqueous solubility [mg·L^−1^]^b^	Odor quality^d^
Geosmin	C_12_H_22_O	182.30	3.7	6.66 × 10^−5^	270	5.49 × 10^−5^	150.2	Musty, earthy, beetroot‐like
2‐MIB	C_11_H_20_O	168.28	3.1	5.76 × 10^−5^	208	6.68 × 10^−5^	194.5	Moldy, musty

^a^ Retrieved from PubChem (nih.gov).

^b^ Retrieved from [18].

^c^ Retrieved from ChemicalBook (predicted).

^d^ Retrieved from OdorantDB.

### 2.2 Sample Preparation

#### 2.2.1 Aqueous Solutions for Fragmentation Studies and Calibrations

The first part of the investigation was conducted using a liquid calibration unit (LCU) (model LCU‐a; IONICON Analytik GmbH, Innsbruck, Austria) as the sample injection method, whereby aqueous samples are vaporized via a nebulizer into a heated evaporation chamber, creating a gas flow into the instrument at defined trace concentrations [19]. Aqueous solutions containing standards of pure geosmin or 2‐MIB were prepared in ultrapure water to final concentrations of 17.28 and 15.95 mg·L^−1^, respectively. Aqueous‐phase concentrations were calculated to achieve gas‐phase concentrations in the LCU evaporation chamber of approximately 100 μg·L^−1^ for each compound. Headspace glass vials of 20 mL were used to contain the solutions. The LCU was operated with a liquid sample flow of 5 μL·min^−1^, with the solutions vaporized into the evaporation chamber (110°C) and then transferred through a heated inlet hose (80°C) into the PTR‐TOF‐MS instrument.

#### 2.2.2 Aqueous Solutions for Static Headspace Measurements

Aqueous solutions of 5 mL containing the target compounds were prepared in 20 mL headspace vials. For the headspace optimization study, 0.5 μg·L^−1^ solutions containing both geosmin and 2‐MIB (50:50 v/v) in methanol were used. Sodium chloride (NaCl), after some preliminary analyses to test solubility limits at room temperature, was additionally added for static headspace measurements at concentrations of 0, 90 and 180 g·L^−1^ and all injections were carried out using an MPS2 autosampler (Gerstel GmbH & Co. KG, Mülheim an der Ruhr, Germany).

#### 2.2.3 Stir‐Bar Sorptive Extraction (SBSE) for GC–MS Validation Analysis

Geosmin quantitation in water by GC–MS analysis was performed using a method adapted from [9]. The method's validation data were compared with those from previous studies reporting the use of the same methodology [6, 27, 28, 29] and metrics were highly comparable (LOD = 0.4 g·L^−1^; LOQ = 1.2 g·L^−1^, linearity (*R* ^2^) = 0.9978 and Relative Standard Deviation (RSD) = 12%). In brief, a commercial stir bar (Twister; Gerstel GmbH & Co. KG, Mülheim an der Ruhr, Germany) coated with polydimethylsiloxane (PDMS; length 2 cm and thickness of 1 mm) was added to 20 mL of the aqueous samples in a 50 mL glass vial and then covered with a plastic lid. Extraction was carried out at room temperature by rotating the stir bar at 1000 rpm for 120 min. After extraction, the stir bar was removed with forceps, rinsed with cold distilled water, dried with a lint‐free tissue, and transferred to thermal desorption tubes. External calibration curves were prepared from a GC‐grade mixture solution (1:1 v/v) of geosmin and 2‐MIB in a dilution series of 0, 50, 100, 250, and 500 ng·L^−1^ in deionized water. All analyses were carried out in triplicates, for both the sample and calibration curve points.

### 2.3 Headspace Optimization Study

Preliminary studies assessed and compared the sensitivities of the analyses via LCU and static headspace injections (see Supporting Information S1: Supplementary Material 2); the latter was chosen for further optimization. The selection of factors and variables to maximize the sensitivity of the method was motivated by the physicochemical relationships described in Equations (1) and (2).

The amount of analyte in the gas phase ($C_{g}$), which has an impact on the detection sensitivity, depends on its concentration in the liquid phase as well as its partition coefficient and phase ratio, as described by Equation (1) [23],

(1) $$A\propto C_{g}=\frac{C_{0}}{K+\beta }$$

where A is the analyte signal intensity (e.g., peak area in GC–MS) in a given volume of headspace, $C_{g}$ is the gas‐phase concentration of the analyte in headspace, $C_{0}$ is the initial concentration of the analyte in the sample, K the partition coefficient, and β is the phase ratio in the static system (e.g., the vial). In this study, optimization factors were only centered on K, which is described by Equation (2) [23],

(2) $$K\propto \frac{1}{{p_{i}}^{{}^{\circ}}\cdot \gamma_{i}}$$

where ${p_{i}}^{{}^{\circ}}$ is the vapor pressure (temperature‐ and time‐dependent) and $\gamma_{i}$ is the activity coefficient (matrix‐composition‐dependent) of the analyte.

Given their relevance in affecting the analyte concentration in headspace, factors selected to be investigated by means of a full factorial design were temperature, time (to maximize ${p_{i}}^{{}^{\circ}}$) and NaCl (to maximize $\gamma_{i}$) [23]. Three levels of each factor were investigated and are detailed in Table 2. NaCl was chosen because it is commonly used in analytical chemistry to promote the release of volatile compounds from aqueous solutions by the *salting out* phenomenon. Furthermore, findings previously reported suggest an increased volatilization potential of microbial metabolites by the addition of salt [10, 31]. Additionally, the detection of low ppt concentrations of the target compounds was maximized by using a rapid headspace injection rate. Injection parameters were selected so that the largest headspace volume (syringe capacity: 2.5 mL) was injected at the fastest rate possible into the instrument. Based on the available conditions, 2.5 mL of the headspace of a 5 mL sample contained in 20 mL vial was injected at a rate of 1 mL·s^−1^, minimizing further dilution by the inlet sample gas flow. Overall, this experimental design implied 27 different experiments, which were performed in randomized order, in triplicates and on different days.

**TABLE 2:** Full factorial design: factors and levels.

Factors	Low level	Middle level	High level	Extra levels	
x_1_: Incubation temperature (°C)	50	70	90	110	130
x_2_: Incubation time (min)	5	10	15	—	—
x_3_: Salt content (g·L^−1^)	0	90	180	—	—

After the first round of experiments, two extra levels for temperature as a factor were further investigated (detailed in Table 2) to explore the limits of the system and method; this implied eight new experimental points, which were also performed in triplicates (105 measurements). In PTR‐MS, high sample humidity increases the concentration of hydrated hydroxonium ions, H_3_O^+^(H_2_O)_n_, which can alter the ion chemistry with the target VOCs and negatively affect the sensitivity towards certain compounds. Several studies have reported the decline in sensitivity for many compounds when humidity was increased, such as benzene and toluene, hydrogen sulfide, and formaldehyde, among others [32, 33].

### 2.4 PTR‐TOF‐MS Analyses

#### 2.4.1 PTR‐TOF‐MS Instruments

The studies reported herein were performed on two different PTR‐TOF‐MS instruments: a PTR‐TOF 8000 and a PTR‐TOF 6000 × 2 (IONICON Analytik GmbH, Innsbruck, Austria). The fragmentation studies using the LCU for direct injection were made using the former, whereas the static headspace analyses were made using the latter.

#### 2.4.2 Fragmentation Studies and Calibrations

The LCU injection studies used a PTR‐TOF‐MS operating with a drift tube temperature of 60°C and with ratios of voltage (U_drift_)/pressure (p_drift_) of 400 V/2.4 mbar, 550 V/2.0 mbar, 550 V/2.2 mbar and 600 V/2.0 mbar, respectively. This resulted in corresponding reduced electric fields E/N of 70, 85, 105 and 125 Td, respectively, whereby 1 Td = 10^−17^ V·cm^−2^. The sampling inlet connecting the LCU with the instrument was kept at 80°C.

#### 2.4.3 Static Headspace Study

Measurements under static headspace conditions were carried out with the PTR‐TOF‐MS drift tube operated at a pressure of 2.6 mbar, a temperature of 80°C, and a voltage (U_drift_) of 269 V (resulting in an E/N of 71 Td). This E/N was found in the fragmentation studies to yield the highest abundance of the dehydration products of geosmin and 2‐MIB and was therefore used in all subsequent analyses. Static headspace injection into the PTR‐TOF‐MS inlet was achieved using the injection port of a GC oven (in the absence of a chromatographic column), which was connected via a 1 mm ID polyether ether ketone (PEEK) inlet capillary to the PTR‐TOF‐MS instrument; both the inlet line and GC oven were kept at 80°C. For the experimental points with incubation temperatures of 110°C and 130°C, the sampling syringe, transfer line, and drift tube were kept at 130°C to avoid cold spots and minimize condensation; all other operating parameters were kept constant.

### 2.5 Data Acquisition and Processing

For the LCU experiments, measurements were made to generate one spectrum every 2 s for the mass range *m/z* 10–207. Background and sample injection spectra were averaged over 200 cycles (400 s). The data were mass‐corrected with respect to two reference ion anchor points (*m/z* 21.022 (H_3_^18^O^+^) and *m/z* 203.943 (C_6_H_5_I^+^)), while an additional ion at *m/z* 330.848 (C_6_H_5_I_2_^+^) was used for the spectra calibration of the static headspace experiments. For the headspace experiments, spectra were collected every 0.5 s for the mass range *m/z* 10–367. Instrument‐specific compound transmission curves were used for both measurement approaches (LCU and static headspace). Signal integration and mass selection were achieved by fitting Gaussian curves to the averaged spectral peaks and automatically generating peak systems that could discriminate the exact *m/z* under investigation using the IONICON Data Analyzer (IDA) software (version 2.2.2.1, IONICON Analytik GmbH, Innsbruck, Austria). Extracted raw data were later normalized to the sum of hydroxonium, monohydrate and dihydrate cluster primary ions via their ^18^O isotopologues to generate normalized counts per second [ncps].

### 2.6 SBSE‐GC–MS Analysis

VOCs adsorbed on the stir bars were desorbed through a two‐step procedure using an automatic thermal desorption unit (TurboMatrix 350, Perkin Elmer, Shelton, CT). First, primary desorption was carried out by heating the stir bar to 240°C for 15 min with a carrier gas flow of 50 mL H_2_·min^−1^. Second, the volatiles desorbed were trapped on Tenax TA held at 1°C and then rapidly heated to 280°C for 4 min to complete the secondary desorption. This resulted in the quick transfer of volatiles from the thermal desorption unit to the GC–MS system (a 8890 GC coupled to a 5977B MSD; Agilent Technologies, Palo Alto, CA) through a temperature‐controlled transfer line held at 225°C. A ZB‐Wax capillary column (30 m × 0.25 mm × 0.5 μm) was used for the separation of the transferred volatiles using H_2_ as a carrier gas at an initial flow rate of 1.4 mL·min^−1^. The GC oven program was set as follows: at 35°C isothermal for the first 10 min, then raised to 240°C at a rate of 8°C/min and held for 10 min. Mass spectra of the separated volatile compounds were generated using standard EI settings (70 eV) and detected via a quadrupole mass spectrometer using hybrid settings that combined scan (SCAN) and selected ion monitoring (SIM) modes. SCAN mode scanned the range *m/z* 15–300, while SIM mode specifically targeted *m/z* 112 for enhanced sensitivity. After data acquisition, chromatograms were processed using the MSD ChemStation software (v.E.02.00, Agilent Technologies). The retention time of geosmin was determined through the calibration curve points and peak areas were calculated based on the SIM chromatograms.

### 2.7 Data Analysis

All data were visualized and further processed using MATLAB R2023b (MathWorks, Natick, MA). For the fragmentation studies via the LCU measurements, a mean of 200 cycles was calculated from the signal. For static headspace injections, the summation of data points containing the peak and background noise (30 cycles) was used as final intensity value (see Supporting Information S1). All statistical analyses and data visualization of the headspace optimization study were performed using JMP Pro, version 18.0.0 (SAS Institute Inc., Cary, NC). For the comparison of quantitations between the two methods, an unpaired student *t*‐test analysis was performed using MATLAB R2023b (MathWorks). This is used to compare how different two means are from unrelated groups (e.g., same samples measured by two different methods).

## 3 Results and Discussion

### 3.1 Fragmentation Pattern Assessments of Geosmin and 2‐MIB

**FIGURE 1:**
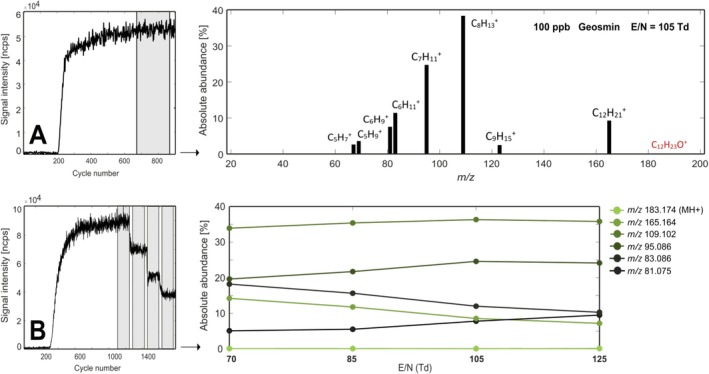
(A) Main product ions of geosmin at E/N 105 Td. (B) Fragmentation pattern of the most abundant ions (> 5% absolute abundance) at four E/N conditions. Absolute abundances are calculated as the percentage [%] of the signal intensity [ncps] of each ion with respect to the sum of intensities of all detected product ions.

**FIGURE 2:**
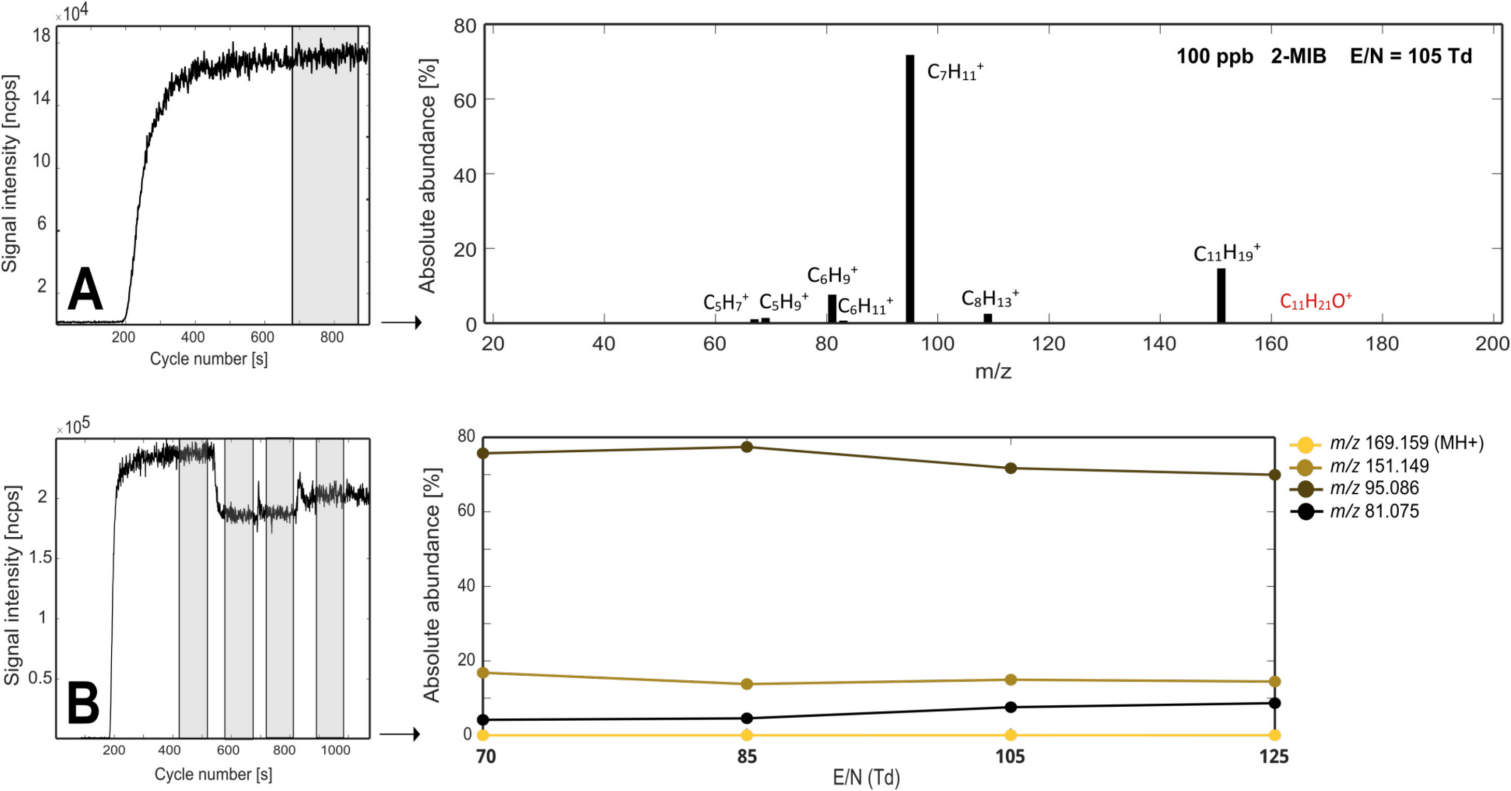
(A) Main product ions of 2‐methylisoborneol (2‐MIB) at E/N 105 Td. (B) Fragmentation pattern of the most abundant ions (> 5% absolute abundance) at four different E/N values. Absolute abundances are calculated as the percentage [%] of the signal intensity [ncps] of each ion with respect to the sum of intensities of all detected product ions.

Geosmin and 2‐MIB underwent dissociative proton transfer reaction under all conditions tested, as observable in Figures 1 and 2, respectively. Geosmin, whose protonated molecule is *m/z* 183.174, always yielded its dehydration product (‐H_2_O) at *m/z* 165.164 (9.3%, C_12_H_21_^+^, 14 ppm), as well as other smaller fragments in greater abundance, such as *m/z* 109.102 (38.4%, C_8_H_13_^+^, 7 ppm), *m/z* 95.086 (24.7%, C_7_H_11_^+^, 52 ppm), and *m/z* 83.086 (11.4%, C_6_H_11_^+^, 35 ppm). 2‐MIB showed a similar behavior, yielding its dehydration product at *m/z* 151.148 (14.6%, C_11_H_19_^+^, 5 ppm), but with the dominant product at *m/z* 95.086 (71.8%, C_7_H_11_^+^, 52 ppm). These observations align with the PTR‐MS data previously reported for these compounds in the literature, as dehydration is typically the only product channel for tertiary and higher alcohols in PTR‐MS [24, 34, 35]. Exceptions have been reported in higher alcohols with neighboring double bonds, such as unsaturated alcohols or phenols, a phenomenon that could be explained due to the resonance stabilization or charge delocalization of the oxygen two lone pairs in a hydroxy group [36, 37].

**FIGURE 3:**
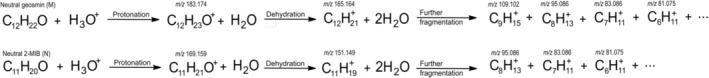
Schematic of the reaction pathways of geosmin and 2‐methylisoborneol upon proton transfer reaction. Hydroxonium ions protonate the hydroxy group of the compounds, leading to a dehydration reaction and further fragmentation.

To examine whether the protonated molecules of both compounds were visible under softer protonation conditions, measurements at four different reduced electric field (E/N) values were performed. E/N is an important parameter because it determines the energy at which proton transfer reactions occur (the greater the energy, the higher the likelihood of a dissociative reaction) as well as the production of H_3_O^+^(H_2_O)_n_ clusters (the greater the energy, the greater the suppression of clustering). As is displayed in Figures 1B and 2B, proton transfer reactions for both geosmin and 2‐MIB generally vary little with E/N (within the tested range). A greater abundance of both dehydration products (*m/z* 165.162 and *m/z* 151.145) is reached at 70 Td and none of the conditions tested yielded the protonated molecule of the two target compounds. Figure 3 illustrates the chemical ionization reaction pathways for both compounds.

The dehydration of geosmin into argosmin, which is odorless, has been reported under different acidic environments, such as hydrochloric [38, 39], citric and acetic acid [33], as well as an acidic algicide [34]. Moreover, several studies have also reported the dehydration of 2‐MIB under acidic conditions, mostly yielding 2‐methylenebornane and 1‐methylcamphene, which are also odorless [40, 42, 43]. As shown, previous findings align with the observations presented in this study. To our knowledge, however, our work represents the first time geosmin and 2‐MIB have been investigated by PTR‐MS. Understanding the ionization pathways of the target compounds upon proton transfer reaction is crucial when aiming to develop analytical methods capable of quantifying these compounds in matrices like water. Hence, this study uses the distinct dehydration products of geosmin and 2‐MIB (*m/z* 165.162 and *m/z* 151.148, respectively) for quantification purposes. Other fragmentation products, like *m/z* 109.101, *m/z* 95.091, *m/z* 83.089, and *m/z* 81.075, besides being common fragments of both compounds in PTR‐MS, are also typical fragments of several other mono‐ and sesquiterpenes [44, 45]. Therefore, these smaller *m/z* were not considered further for the quantification method subsequently developed in this study (see Section 3.2), as mass interferences and thus quantification over and/or underestimations are deemed more likely.

### 3.2 Headspace Sensitivity Optimization Study

**FIGURE 4:**
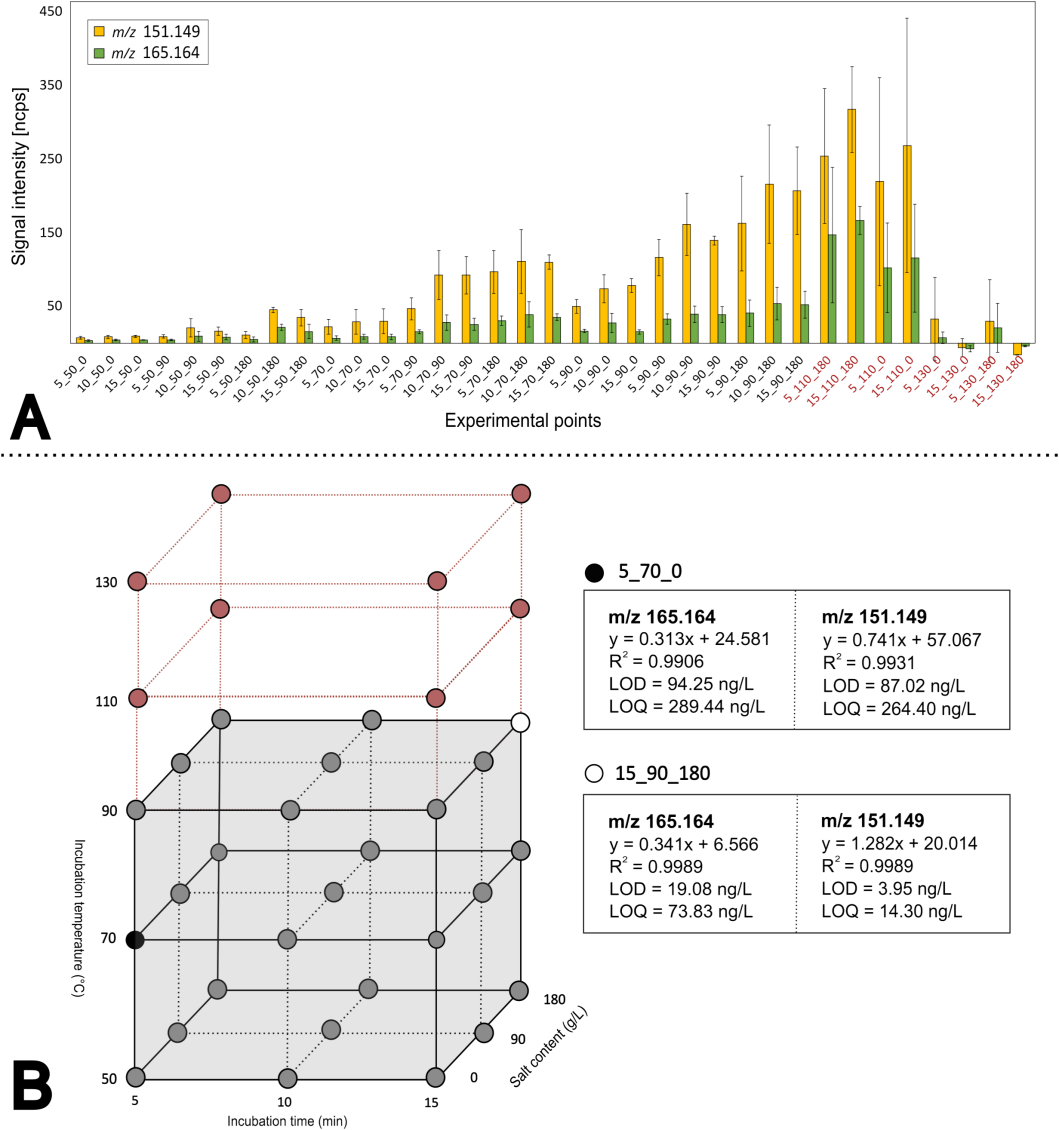
(A) Experimental data of the factorial design for all 35 different experiments. Experiments are labelled by the code: *Incubation time_Incubation temperature_Salt content* (i.e., 10_70_0 corresponds to an experimental point where incubation time was of 10 min, incubation temperature at 70°C and NaCl content of 0 g·L^−1^). (B) Comparison between nonoptimized (5_70_0, black circle) vs. optimized (15_90_180, white circle) static headspace methods and their sensitivities (linearity, LOD, and LOQ). The extra temperature levels studied are also displayed and colored red as these data points were not modeled.

The injection data from all static headspace experiments included in the factorial design are displayed in Figure 4A. The incubation temperature clearly led to an increase in signal intensity, with the exception of the incubation temperature at 130°C, which has a negative impact on the signal. The measurement error for the experiments at an incubation temperature of 110°C was considered too high (CV^1^ ≈ 40%). These observations led to an exclusion of the experimental points with incubation temperatures of 110°C and 130°C. Weighted least squares (WLS) regression was used to model the data of the remaining 27 experimental points (grey‐colored experimental cube in Figure 4B) to gain quantitative insights on the effect of the different factors studied. WLS is a parametric modeling method that minimizes the sum of squares of the residuals to estimate the linear parameters ($\beta_{i}$) associated with the main factors and their interactions involved for a given response *Y*. The model is mostly used when residuals show heteroskedasticity (nonconstant variance) as observations are weighted based on this nonhomogeneity. After a comparison between ordinary least squares (OLS) and WLS, the latter was selected as the best fitting modeling method (for more details see Supporting Information S1: Supplementary Material 4). The WLS model is specified in Equation [3] and results are displayed in Table 3.

(3) $$\begin{array}{c} y=\beta_{0}+\beta_{1}x_{1}+\beta_{2}x_{2}+\beta_{3}x_{3}+\beta_{4}x_{1}x_{2}+\beta_{5}x_{1}x_{3}+\beta_{6}x_{2}x_{3}+\beta_{7}x_{1}x_{2}x_{3}+e \end{array}$$

where $x_{1}$, $x_{2}$, and $x_{3}$ are the main factors investigated (incubation temperature, incubation time and salt content), $x_{1}x_{2}$, $x_{1}x_{3}$, $x_{2}x_{3}$, $x_{1}x_{2}$, and $x_{1}x_{2}x_{3}$ are the possible interactions (2nd and 3rd order), y is the measured response (signal intensity [ncps]), $\beta_{0}$, $\beta_{1}$, $\beta_{2}$ $\beta_{3}$, $\beta_{4}$, $\beta_{5}$, $\beta_{6}$, and $\beta_{7}$ are the model coefficients, and e represents the residuals.

**TABLE 3:** Estimated parameters, standard error and *p*‐value of the different factors investigated.

		*m/z* 165.162			*m/z* 151.148		
Factors	Coefficients	Estimate	Std error	*p*	Estimate	Std error	*p*
Incubation temperature (°C)	β_1_	11.76	0.74	**< 0.0001** *	51.98	3.04	**< 0.0001** *
Incubation time (min)	β_2_	2.50	0.67	**0.0003** *	10.49	3.06	**0.0010** *
Salt content (g·L^−1^)	β_3_	10.69	0.75	**< 0.0001** *	35.46	3.40	**< 0.0001** *
Temperature*time	β_4_	−1.52	0.78	0.0543	2.11	3.18	0.0508
Time*salt	β_5_	2.37	0.79	**0.0036** *	1.83	3.55	0.6085
Temperature*salt	β_6_	5.91	0.87	**< 0.0001** *	23.67	3.51	**< 0.0001** *
Temperature*time*salt	β_7_	−1.08	0.91	0.2439	−5.36	3.72	0.1541
Intercept	β_0_	20.50	0.64	**< 0.0001** *	71.70	2.97	**< 0.0001** *

* Denotes statistically significant values (*p* < 0.05).

Incubation temperature was the leading factor (highest estimated parameters in both models) for increasing the headspace sensitivity of the method for both products monitored by PTR‐MS. This was expected, as temperature is a key parameter that increases the vapor pressure of volatile compounds in both open and closed systems [23]. NaCl was the second most influential factor within the experimental design. The addition of NaCl and other inorganic salts is a common procedure in analytical methods to induce *salting out*. Moreover, the addition of large amounts of salt increases the sample volume, decreasing the phase ratio between the liquid and gas compartments. This can also boost the headspace sensitivity and cannot be ignored [23]. The interaction between these two factors, temperature and salt content, also significantly affected the measured signal intensity of both products. Solubility of NaCl slightly increases with temperature, which may explain why this interaction effect is observed for both products. This is key information that can be studied by such an experimental design, as factors (or variables) do not always act additively towards a given response [39]. Incubation time also significantly affected the measured response for *m/z* 151.148 (not for *m/z* 165.162). Nevertheless, incubation time contributes least to the signal response of both products. In technical terms, incubation time could be referred to as equilibration time, which is known to vary according to sample volume and compound solubility. Given the fixed phase ratio β and the relatively low solubility of these compounds in water (150.2 mg·L^−1^ for geosmin and 194.5 mg·L^−1^ for 2‐MIB), with correspondingly low partition coefficients (K), it is a logical consequence that the observed effect of time in the measured response was not as large as with the other factors studied.

After gaining a better understanding of the factors affecting signals of the target products in the static headspace measurements, the expected increased sensitivity of the static headspace method was tested under the optimal conditions (15_90_180) versus a nonoptimized experimental point (5_70_0). Optimal conditions were chosen by maximization, as specified in Supporting Information S1: Supplementary Material 3. Calibration curves with 0, 5, 10, 25, 50, 100, 250, and 500 ng·L^−1^ of geosmin and 2‐MIB were made in triplicates. Results are displayed in Figure 4B. The limits of detection and quantitation (LOD and LOQ, respectively) of the optimized method for both compounds substantially decreased, with an LOD of 19 ng·L^−1^ for geosmin (detected at *m/z* 165.162) and 4 ng·L^−1^ for 2‐MIB (detected at *m/z* 151.148), respectively.

The responses at incubation temperatures of 110°C and 130°C did not follow the expected progression, with highly variable intensities observed for 110°C and almost no signal observed at 130°C. The latter could partly be attributed to the increased sample humidity under these conditions, whereby a significant increase in the primary water cluster was observed during injection (see Supporting Information S1: Supplementary Material 4). To demonstrate this, the ^18^O isotopologues *m/z* 21.022 (H_3_^18^O^+^) and *m/z* 39.033 (H_3_^18^O(H_2_O)^+^) and the ^16^O isotopologue at *m/z* 55.039 (H_3_^16^O(H_2_O)_2_^+^) were monitored across all temperatures for both 0 and 180 g·L^−1^ NaCl conditions. Here, it was observed that the increases of mono‐ and dihydrate clusters were significant after incubation at higher temperatures. It is not clear, however, why the signal at 110°C was relatively prominent compared to an almost zero signal at 130°C. The humidity dependence in PTR‐MS measurements to different VOCs is well documented and has been observed to vary greatly depending on the compound's chemical properties, as well as the PTR‐MS operating conditions [32, 33]. It is hypothesized that besides the negative effect of water vapor during the reaction, the physical accumulation of condensed water vapor in the headspace vials—which occurs more rapidly and dominantly at 130°C—could have impeded the extraction of volatilized geosmin and 2‐MIB in the headspace. These observations highlight the experimental boundaries to the method.

### 3.3 Comparison Between the Rapid PTR‐MS Approach With a Benchmark GC–MS Method on RAS Samples

To determine the effectiveness of the method and to compare it analytically with a benchmark procedure, a set of five RAS water samples with unknown concentrations of geosmin, one control and four spiked samples, were analyzed using the two different approaches. 5‐point calibration curves (in triplicate) were used to predict the concentration of geosmin in the RAS samples (also measured in triplicate) using both methodologies (a total of 60 measurements). The rapid static headspace method using PTR‐TOF‐MS was selected (incubation time: 5 min), with a SBSE‐GC–MS method developed with the purpose of quantifying low concentrations of geosmin in water employed as a benchmark. The latter was adapted from [9], whereby extraction conditions were retained but the use of longer stir bars (2 cm long) and a larger sample volume (20 mL) were applied to increase sensitivity (see Section 2.2.3). Given the low relevance of incubation time in the headspace sensitivity of the PTR‐MS method (see Section 3.3), the shortest incubation time was selected to shorten analytical time. Specifications of both methods are outlined in Table 4.

**TABLE 4:** Technical specifications of each of the analytical methods compared: PTR‐MS and SBSE‐GC–MS.

Specifications	Static headspace PTR‐TOF‐MS	SBSE‐GC–MS
Incubation/extraction time (min)	5	120
Incubation temperature (°C)	90	RT^a^
Agitation (yes/no)	Yes	Yes
NaCl (yes/no)	Yes	No
Sample volume (mL)	5	20
MS	TOF (SCAN)	QMS (SIM *m/z* 112)
Separation/detection time (min)	< 0.1	30
Total analysis time per sample (min)	≈5–6	≈150

^a^ RT: Room temperature (20**°**C–25**°**C).

**FIGURE 5:**
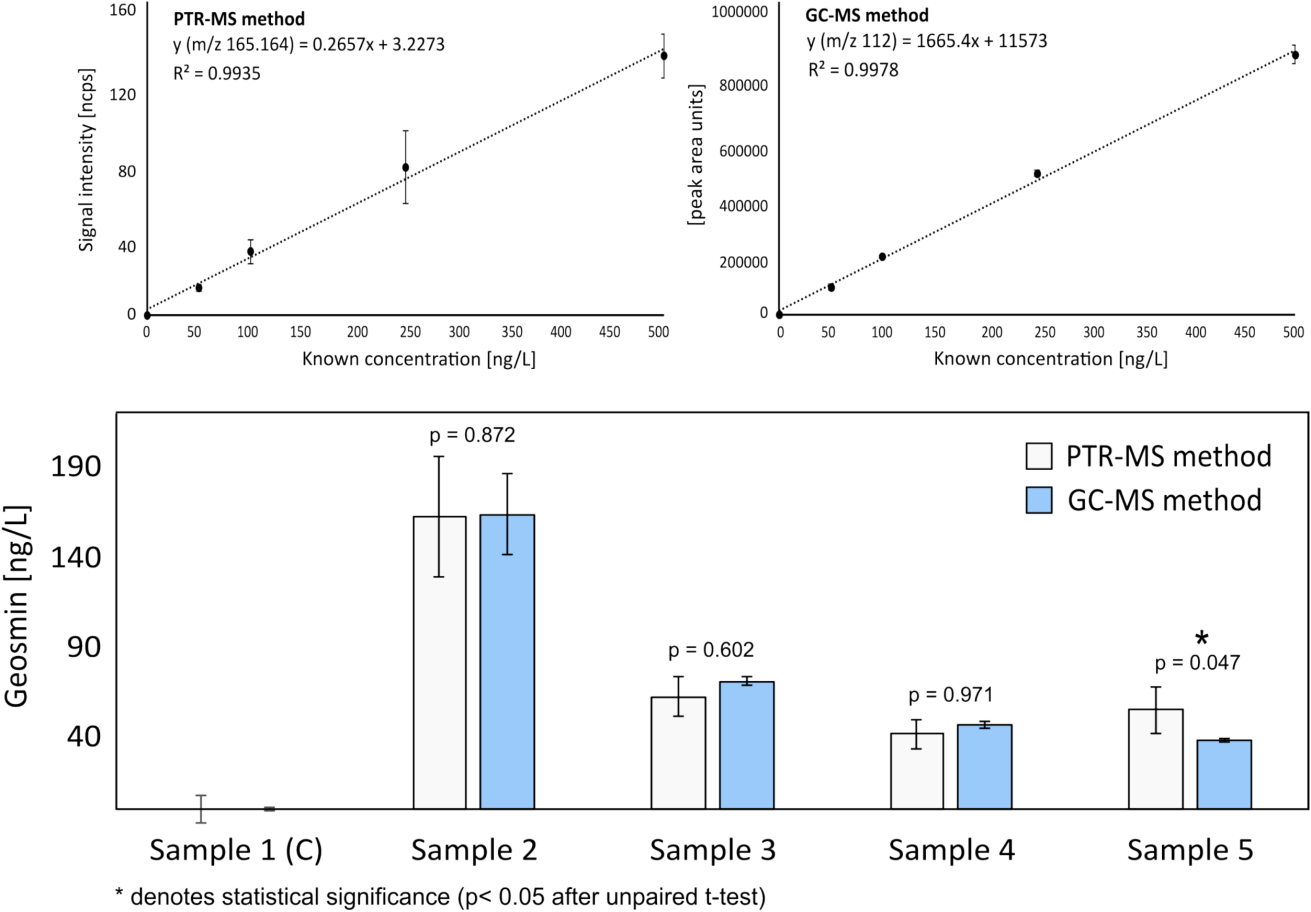
Comparison of the calibration curves obtained via both methods (top) as well as the corresponding quantification of geosmin (bottom). Significant differences per sample (*p* < 0.05) between the results obtained with each method employed are marked with an asterisk (*).

Both methods yielded comparable results across samples (Figure 5). Three (samples 1–4) out of the four geosmin concentrations quantified in the spiked samples were not statistically different (unpaired student t‐test). This should encourage improvement to achieve better analytical qualities (higher accuracy, precision, sensitivity, and robustness). Even though the PTR‐MS method is approximately 25–30 times faster than its GC–MS counterpart, speed comes with compromises in sensitivity and analytical variability (poorer repeatability). The CV of the analytical replicates measured with PTR‐MS had a mean of approximately 20% across samples, while being 5% for the GC–MS analyses.

The detection limits of the developed static headspace PTR‐MS analysis method were within similar ranges to the odor thresholds of the two off‐odor target compounds, with an LOD below OT for 2‐MIB (4 ng·L^−1^ LOD vs. 15 ng·L^−1^ OT but an LOD above OT for geosmin (19 ng·L^−1^ LOD vs. 4 ng·L^−1^ OT)). Consequently, the method represents a prospective screening approach to detect the onset of off‐flavors spikes during production or under different experimental conditions. It is noteworthy to mention that this should be deemed as a method under development, considering the complexity in the lifecycle of analytical methods. Further validation procedures, including interlaboratory and revalidation procedures, would be the next steps to further develop this approach for prospective routine analysis [40]. Moreover, several further improvements could be considered as future developments to achieve sub‐ng·L^−1^ sensitivities. Besides instrumental sensitivity, sample preparation variables, such as headspace vial capacity, syringe capacity, sample volume, as well as the use of other salts, could be optimized to further refine the sensitivity of the PTR‐MS method. The notable higher sensitivity for 2‐MIB over geosmin, arguably, can be due to differences in the compounds' volatility as well as instrumental response factor. 2‐MIB is slightly more volatile and less sticky than geosmin, which may translate into a more efficient headspace build‐up and generally a cleaner and thus greater instrumental response.

Headspace analyses coupled with PTR‐MS have long been used as a targeted approach to detect aroma compounds in different aqueous matrices [1, 2], albeit mainly for the detection of compounds at concentrations in the ppb range or higher. For the analysis of trace compounds dissolved in water, [43] developed a direct aqueous injection analysis coupled to PTR‐MS that achieved detection limits of 100 ng·L^−1^ for pyridine (*m/z* 80). However, more sensitive methods are required for the detection of aroma compounds with extremely low OTs in water, such as geosmin and 2‐MIB, as well as earthy‐smelling pyrazines, such as 2‐isobutyl‐3‐methoxypyrazine or 2‐isopropyl‐3‐methoxypyrazine, or haloanisoles, fenchol and fenchone [7, 21, 22]. The results presented here therefore unlock the potential of static headspace PTR‐MS via fast injections to measure low concentrations of undesired off‐odor metabolites in water.

## 4 Conclusions

Rapid chemical analytical methods are demanded across industries to reduce costs and offer a high sample throughput to provide a quicker screening of product quality. In the aquaculture industry, GC–MS is the current method of choice to quantify the prominent off‐odors geosmin and 2‐methylisoborneol in water. Although GC–MS offers high sensitivity, sample analysis can be laborious and time‐consuming. This study proposes the use of PTR‐MS as an alternative, faster method for the quantification of these two analytes in water. Optimized static headspace fast injection by PTR‐TOF‐MS yielded comparable results to an established SBSE‐GC–MS benchmark method. This study paves the way for the development of similar analytical methods based on fast injection headspace PTR‐MS detection for rapid monitoring of other relevant (undesired) metabolites in water. For the aquaculture industry, the availability of high‐throughput methods could help streamline off‐flavor detection in operations, achieve more continuous product quality monitoring, and support large‐scale screening aimed at mitigating the presence of these metabolites. Ideally, an online approach for the in situ detection of these compounds at the operations would be highly advantageous for RAS producers. The implementation of these methods in commercial operations is feasible yet is still associated with high costs of the initial investment, operation, and highly skilled human labor, which presents a challenge that must be overcome.

## Author Contributions

**Pedro Martínez Noguera:** conceptualization, investigation, methodology, validation, formal analysis, data curation, writing – original draft, writing – review and editing, visualization. **Sylvester Holt:** conceptualization, methodology, writing – review and editing. **Raju Podduturi:** methodology, writing – review and editing. **Wender L. P. Bredie:** writing – review and editing. **Jonathan D. Beauchamp:** conceptualization, methodology, writing – review and editing. **Mikael A. Petersen:** conceptualization, methodology, writing – review and editing.

## Funding

This research was funded by the European Union's Framework Program for Research and Innovation Horizon 2020 under the Marie Sklodowska‐Curie Training Network RASOPTA Grant Agreement No. 956 481. Part of the presented data were generated through accessing research infrastructure funded by FOODHAY (Food and Health Open Innovation Laboratory, Danish Roadmap for Research Infrastructure).

## Conflicts of Interest

The authors declare no conflicts of interest.

## Supporting information


**Data S1:** Supporting information.

## Data Availability

The data that support the findings of this study are available from the corresponding author upon reasonable request.

## Endnotes

^1^
Coefficient of variation (CV, expressed in %) is defined as the ratio between the standard deviation and the mean of a set of measurements or data points.
