# Supplementary material for: Fast and Sensitive Measurement of Off‐Flavors in Recirculating Aquaculture Systems
Source: Rapid Commun Mass Spectrom. 2026 May 1;40:e70085. doi: 10.1002/rcm.70085 (PMC13133655; doi:10.1002/rcm.70085)
Supplement: Supplementary file 1 — Data S1: Supporting information. [file RCM-40-e70085-s001.docx]

**SUPPLEMENTARY MATERIAL**

**Fast and Sensitive Measurement of Off-Flavors in Recirculating Aquaculture Systems**

Pedro Martínez Noguera^1^, Sylvester Holt^1^, Raju Podduturi^1^, Wender L.P. Bredie^1^, Jonathan Beauchamp^2^, Mikael A. Petersen^1^

^1^Design and Consumer Behaviour, Department of Food Science, University of Copenhagen, Rolighedsvej 26, 1871 Frederiksberg, Denmark.

^2^Department of Sensory Analytics and Technologies, Fraunhofer-Institute for Process Engineering and Packaging IVV, Giggenhauser Str. 35, 85354 Freising, Germany

## **Supplementary material 1**

### **Fast injection peak data processing**

The signal response [ncps] recorded after raw data normalization of every fast injection was numerically expressed as the summation of all data points contained within a 30-cycle window, which included both the injection peak and background noise before and after the injection (See SM1).

**SM 1**. The scheme of the fast injection of a given mass (on the left), where after recording the background noise the injection occurs in 5 cycles (2.5 s). A processing window of 30 cycles (15 s) and the summation of all data points therein have been used to numerically express the signal response of these fast injection peaks (on the right).

## **Supplementary material 2**

### **Initial sensitivity assessment of LCU and static headspace methods before optimization**

Before carrying out the optimization study presented in Section 3.2, preliminary tests were performed with both injection methods (LCU and fast injection static headspace) to check for general instrumental sensitivity values towards both targeted masses (*m/z* 165.162 and *m/z* 151.145). However, they were performed with two different PTR-MS instruments, PTR-MS 8000 and PTR-MS 6000x2, respectively, and thus a direct comparison is not intended as the instrumental sensitivities can differ significantly. However, this initial testing is thought useful to motivate why the static headspace PTR-MS method was selected, given its room for optimization

To perform these tests, dilution steps with a LCU were performed “online” by mixing the sample solutions with pure water at different ratios (See SM2 A). However, an improved transfer (and thus sensitivity) from the vaporized liquid to the instrument is not possible given the predefined parameters which a LCU is typically operated with. Calibration curves using the fast injection static headspace method with aqueous solutions with different known concentrations of geosmin and 2-MIB were also obtained (See SM2 B). In this specific case the measured signal intensity could be optimized with a commercial autosampler, where factors such as temperature, time, headspace volume, etc. can be modified towards signal maximization.

**SM2**. A: Dilution steps of 100 ppb solutions of geosmin and 2-MIB at E/N = 105 Td with an LCU and a PTR-MS model 8000. B: Series of fast SH injections of solutions at low ppt concentrations of geosmin and 2-MIB at E/N = 70 Td with a PTR-MS model 6000x2.

To assess the methods’ sensitivity towards the fragments of interest, limit of detection (LOD) as in Eq. [3] and limit of quantification (LOQ) as in Eq. [4] were calculated.

$Y_{LOD}= Y_{blank}+3S_{blank}$ [3]

$Y_{LOQ}= Y_{blank}+10S_{blank}$ [4]

Where $Y_{blank}$ is the average signal intensity [ncps] of a set of blank injections (pure water in triplicates), $S_{blank}$ the standard deviation of the set of blank injections, $Y_{LOD}$ the signal intensity [ncps] of the limit of detection and $Y_{LOQ}$ the signal intensity [ncps] of the limit of quantification.

On the one hand, the LCU method linear accuracy noted by the coefficient of determination (R^2^) of both prediction lines was 0.9997 and 0.9996 for *m/z* 165.162 and *m/z* 151.148 respectively. LOD and LOQ values are generally in the low ppb range, except for the LOD for *m/z* 151.148, which is well into the ppt range (160 ppt). However, as the aim of the study is to develop a method able to predict concentrations in the low ppt range, the LCU method was deemed unsuitable for this specific application. On the other hand, the fast injection static headspace method (70 °C, 5 min and 0 g·L^-1^ NaCl) yielded also promising results in terms of linearity and repeatability. Figure SM2 B represents only one sequence of injections for illustration purposes for the sake of simplicity, but measurements were performed in triplicates and linear regression parameters were calculated based on the average values of each concentration point (data not shown). Coefficients of determination (R^2^) were 0.9874 and 0.9943 for *m/z* 165.162 and *m/z* 151.148 and LODs for both fragments is the low ppt range already (84 and 87 ppt). This finding, together with the fact that the static headspace method could be optimized steered the study to continue using a static headspace fast injection method for the quantification of low concentrations of geosmin and 2-MIB in water.

## **Supplementary material 3**

### **Least squares modelling**

Weighted least squares (WLS) regression was used to estimate the parameters of a linear model (Eq. [3]) of the experimental data included in the full factorial design (See Figure 4B). After the first model tests with ordinary least squares (OLS), even though the relationship between signal measured and predicted was rather linear, the model residuals showed a noticeable heteroskedasticity. Therefore, weighted least squares (WLS) regression using the reciprocal of the variance $(\frac{1}{\sigma_{i}^{2}})$ (where $i$ corresponds to each experimental point) as weighting variable was used to test if the quality of the model improved. The results can be visualized in SM3 A. First, the Root Mean Square Error (RMSE) values of the OLS models for both fragments are considerably higher (8.936 and 27.924 for *m/z* 165.162 and *m/z* 151.148, respectively) than those obtained by WLS (1.082 and 1.377 for *m/z* 165.162 and *m/z* 151.148, respectively). Second, R^2^ values show a better linearity when the data is modelled by WLS compared to WLS (0.91 vs 0.75 for *m/z* 165.162 and 0.93 vs 0.84 for *m/z* 151.148). For these reasons, WLS regression was chosen over OLS regression to model the experimental data (best model fit). In short, given the similar magnitude and direction of the estimated coefficients, smaller RMSE values and better linearity. The modelling results presented in Table 3 and below in Figure SM3 B are therefore the output of the WLS regression model.

To numerically find the optimum experimental point within the experimental design to maximize the method’s sensitivity, instead of interpreting the modelling results and arriving oneself to the best combination of variables towards a given output, this study used the desirability profiling and optimization tools developed by JMP® for that purpose. When multiple continuous factors like time, temperature and NaCl content need to be optimized, the software uses a gradient algorithm and an overall desirability function ($D$, Eq. [5]), which is constructed out of each individual desirability functions ($d_{1}$, $d_{2}$ and $d_{3}$), to optimize a given multivariate response (signal intensity [ncps]).

$D=d_{1}^{\frac{1}{k}}\cdot d_{2}^{\frac{1}{k}}\cdot d_{3}^{\frac{1}{k}}$ [5]

The red dotted lines in Figure SM3 B, both for the predicted response (top) as well as for the desirability functions (bottom), show the convergence of the maximization function at the maximum of each of main factors investigated. It was therefore concluded that the optimal method was 15_90_180, which outputs a predicted signal response of 51.14 and 191.88 for *m/z* 165.162 and *m/z* 151.148, respectively, and overall desirability values of 0.62 for *m/z* 165.162 and 0.60 for *m/z* 151.148.

**SM3**. **A**. Visual comparison between OLS and WLS regressions of the experimental data from each fragment (m/z 165.162 and m/z 151.148). On the left side of each column, the models and their specifications (RMSE, R^2^ and p value). On the right side of each column, the standard (top) and studentized residuals (bottom) of the models across experiments. **B**. Prediction profiler (top first three columns), desirability maximization functions of the factors investigated (bottom first three columns) and maximization function convergence points (red dotted lines).

## **Supplementary material 4**

### **Water cluster ions and incubation temperatures**

To further understand the sample humidity conditions under the different incubation temperatures tested, water cluster-related masses were monitored. Besides *m/z* 21.0227 (H_3_^18^O^+^) to check for stable ionization conditions, we followed ^18^O isotopologue at *m/z* 39.0298 (H_3_^18^O(H_2_O)^+^), as it was clearly visible, and the ^16^O isotopologue at *m/z* 55.0383 (H_3_^16^O(H_2_O)_2_^+^), as its ^18^O isotopologue was not found. The results in ncps were expressed numerically after summating all data points contained within 100-cycle window, which included both the injection peak and background noise before and after the injection.

**SM4**. Signal intensities [ncps] recorded for m/z 21.0227 (H_3_^18^O^+^) (left), m/z 39.0298 (H_3_^18^O(H_2_O)^+^) (middle) and m/z 55.0383 (H_3_^16^O(H_2_O)_2_^+^) (right) across temperatures (50, 70, 90, 110 and 130 °C) and NaCl content extreme points, namely 0 (top) and 180 g·L^-1^ (bottom). Also, only the two extreme points of the incubation times tested (5 (in blue) and 15 min (in orange)) have been plotted.
